# Evaluating a combination treatment of NK cells and reovirus against bladder cancer cells using an in vitro assay to simulate intravesical therapy

**DOI:** 10.1038/s41598-024-56297-7

**Published:** 2024-03-28

**Authors:** Yuree Lim, Jeehun Park, Joung Eun Lim, Minji Park, Seung Kwon Koh, Mijeong Lee, Sang-ki Kim, Seung-Hwan Lee, Ki-Hoon Song, Dong Guk Park, Hyun-Young Kim, Byong Chang Jeong, Duck Cho

**Affiliations:** 1 Department of Biopharmaceutical Convergence, Sungkyunkwan University (SKKU), Suwon, Korea; 2 Department of Molecular Bioscience, College of Biomedical Science, Kangwon National University, Chuncheon, Korea; 3 Department of Urology, Samsung Medical Center, Sungkyunkwan University School of Medicine, Seoul, South Korea; 4 Department of Health Sciences and Technology, SAIHST, Sungkyunkwan University, Seoul, South Korea; 5 Department of Companion & Laboratory Animal Science, Kongju National University, Yesan, Korea; 6 Department of Biochemistry, Microbiology and Immunology, Faculty of Medicine, University of Ottawa, Ottawa, ON Canada; 7 ViroCure Inc., Seoul, Republic of Korea; 8 Department of Surgery, School of Medicine, Dankook University, Cheonan, South Korea; 9 Department of Laboratory Medicine and Genetics, Samsung Medical Center, Sungkyunkwan University School of Medicine, 81, Irwon-Ro, Gangnam-Gu, Seoul, 06351 Republic of Korea

**Keywords:** Immunology, Tumour virus infections, Urological cancer

## Abstract

Intravesical treatment using either reovirus or natural killer (NK) cells serves as an efficient strategy for the treatment of bladder cancer cells (BCCs); however, corresponding monotherapies have often shown modest cytotoxicity. The potential of a locoregional combination using high-dose reovirus and NK cell therapy in an intravesical approach has not yet been studied. In this study, we evaluated the effectiveness of reoviruses and expanded NK cells (eNK) as potential strategies for the treatment of bladder cancer. The anti-tumor effects of mono-treatment with reovirus type 3 Dearing strain (RC402 and RP116) and in combination with interleukin (IL)-18/-21-pretreated eNK cells were investigated on BCC lines (5637, HT-1376, and 253J-BV) using intravesical therapy to simulate in vitro model. RP116 and IL-18/-21-pretreated eNK cells exhibited effective cytotoxicity against grade 1 carcinoma (5637 cells) when used alone, but not against HT-1376 (grade 2 carcinoma) and 253J-BV cells (derived from a metastatic site). Notably, combining RP116 with IL-18/-21-pretreated eNK cells displayed effective cytotoxicity against both HT-1376 and 253J-BV cells. Our findings underscore the potential of a combination therapy using reoviruses and NK cells as a promising strategy for treating bladder cancer.

## Introduction

Bladder cancer is one of the most prevalent malignancies globally, with non-muscle-invasive bladder cancer (NMIBC) constituting approximately 70–75% of all cases^1,2^. The majority of patients with NMIBC undergo a procedure known as transurethral resection of the bladder tumor (TURBT). Following this, intravesical therapy has emerged as a significant therapeutic approach that targets the bladder's inner lining while minimizing the risk of collateral damage to adjacent tissues^3,4^. Characterized by the direct administration of therapeutic agents, including immunotherapeutic agents and chemotherapeutic compounds, into the bladder via catheterization, intravesical therapy is a fundamental element of treatment and is administered for a specified duration of approximately 2 h. This allows immunotherapy, including substances such as Bacille Calmette–Guerin (BCG), to reach and interact effectively with the bladder interior lining^3,4^. This unique strategy takes advantage of targeted drug delivery, enabling higher drug concentrations in the bladder over a brief period when employing combination therapies such as BCG with the immune check point inhibitor nivolumab (ClinicalTrials.gov Identifier: NCT03519256)^5–7^.

Reoviruses, non-enveloped, double-stranded RNA viruses with a diameter of 70–100 nm, specifically target RAS-activated cancer cells^8,9^. A previous study identified and characterized RP116, a reovirus variant isolated from persistently infected HT1080 human fibrosarcoma cells. Although RP116 exhibits truncated σ1, it still demonstrates high oncolytic activity in human colon cancer models^10^. Reoviruses have been widely investigated for their natural oncolytic activity against various types of cancers^11^. Bladder cancer, which is known to involve (upregulated) Ras signaling, has been identified as a target candidate for reovirus therapy^12–15^. In clinical trials, reovirus concentrations have undergone rigorous evaluations, leading to optimal therapeutic outcomes while ensuring safety, even at high doses^16,17^. Therefore, reoviruses are promising candidates for intravesical therapy when directly injected at a short, high dose into the bladder.

Natural killer (NK) cells, a type of cytotoxic lymphocyte, can be considered an alternative agent for intravesical therapy. Expanded NK (eNK) cells isolated from donor blood are considered a promising strategy for cancer treatment and can be used as off-the-shelf drugs because they rarely cause graft-versus-host reactions or disease^18,19^. Additionally, treatment with IL-18/-21 enhances NK cell cytotoxicity, thereby highlighting its potential to promote anti-tumor effects^20,21^. In a recent clinical trial, locoregional high-dose NK cells following four cycles of hepatic arterial infusion chemotherapy (HAIC) with 5-fluorouracil and cisplatin were administered to patients with HCC via hepatic arterial infusion. The results showed excellent therapeutic efficacy and safety^22^. Hence, locoregional high-dose NK cell therapy appears promising for bladder cancer in conjunction with intravesical therapy.

In this study, we investigated the effectiveness of reoviruses and eNK cells as potential therapeutic strategies for bladder cancer. To estimate their efficacy, we designed an in vitro experiment simulating intravesical therapy, involving short-duration and high-concentration treatments. Our findings demonstrate the therapeutic potential of mono- or combined therapy with reoviruses and eNK cells in three different grades of BCC lines. Finally, we propose that high-dose combination therapy with reoviruses and eNK cells may be an effective treatment strategy for bladder cancer.

## Results

### Evaluating cytotoxicity of reoviruses, RC402 and RP116, on bladder cancer cell lines

**Figure 1:**
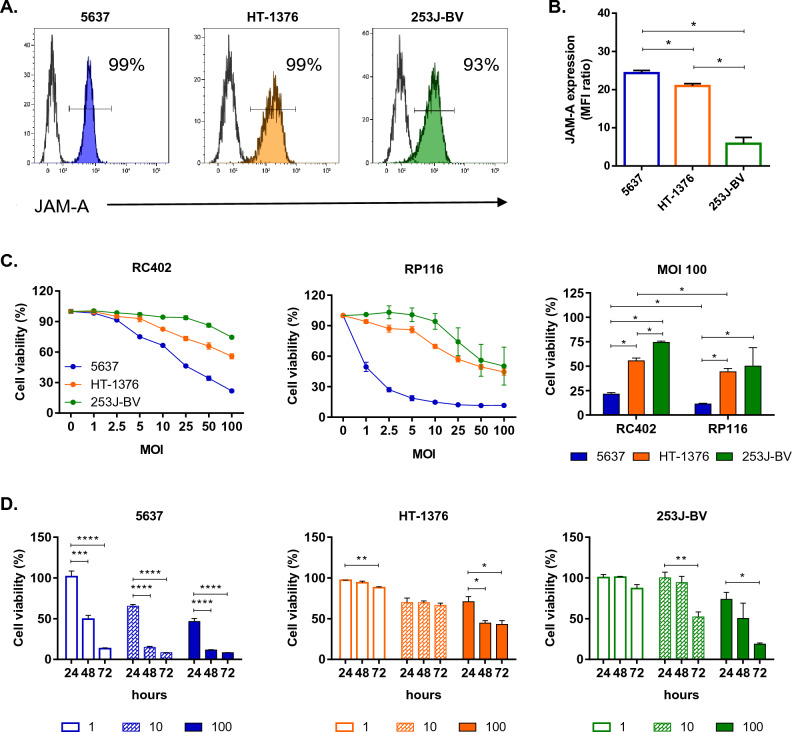
Viability of bladder cancer cell lines to reoviruses infection, RC402 and RP116. (**A**, **B**) JAM-A surface expression on BCC lines was assessed via flow cytometry, and the results are presented as the mean fluorescence intensity (MFI) ratio based on triplicate values in a representative experiment (n = 3). BCC lines were incubated in the presence of reovirus RC402 and RP116 at different MOI values. (**C**) Following a 48 h incubation, cell survival was analyzed by MTS assay, and the mean cell viability was calculated for each group at 100 MOI (right). (**D**) RP116 was treated on BCC lines for 24, 48 and 72 h at various MOI values. All data are shown as means from triplicate independent experiments (5637, HT-1376, n = 3; 253J-BV, n = 2). Statistical significance was calculated by Mann–Whitney test (**A**, **B**) and one-way ANOVA with Turkey’s multiple comparisons test in (**C**). *MOI* multiplicity of infection. *p < 0.1; **p < 0.01; ***p < 0.001; ****p < 0.0001.

Junction adhesion molecule-A (JAM-A) is an integral tight junction protein and has been identified as a reovirus receptor, facilitating intracellular viral infection^23^. To investigate the potential efficacy of reovirus in BCC lines, we assessed the expression of JAM-A in 5637, HT-1376, and 253J-BV using flow cytometry. All three types of BCC lines exhibited more than 90% JAM-A expression (Fig. 1A). When comparing the relative mean fluorescence intensity (rMFI) ratios with the control group, 5637 displayed the highest rMFI ratio, while 253J-BV exhibited the lowest (Fig. 1B). These findings suggest the suitability of these cell lines for reovirus-related investigations.

To evaluate the cytotoxicity of reovirus against bladder cancer cells, we treated three BCC lines, 5637, HT-1376, and 253J-BV, with two reoviruses, RC402 and RP116, at various MOI levels ranging from 1 to 100. The results demonstrated a dose-dependent cytotoxic effect of both reoviruses on all BCC lines after 48 h of treatment. Notably, in the 100 MOI group, the viability of 5637 cells was the lowest, which exhibited the highest JAM-A rMFI ratio (Fig. 1C). Additionally, we confirmed that RP116 showed higher cytotoxicity against BCC lines compared to RC402, leading us to select RP116 for subsequent experiments. Upon analyzing viability across different cell lines, RP116 demonstrated an anti-tumor effect on all cell lines. In particular, RP116 induced a remarkable reduction of 5637 cell viability, resulting in over 90% cell death at 1 MOI over 72 h. Furthermore, the viability of HT-1376 and 253J-BV cells were reduced to less than 50% at 100 MOI for 72 h (Fig. 1D). Based on these results, we confirmed the anti-tumor effect of reoviruses on BCC lines, and RP116 showed dose- and time-dependent cytotoxicity effects on these cell lines.

### Measuring the oncolytic activity of reovirus in an in-vitro assay emulating intravesical therapy

**Figure 2:**
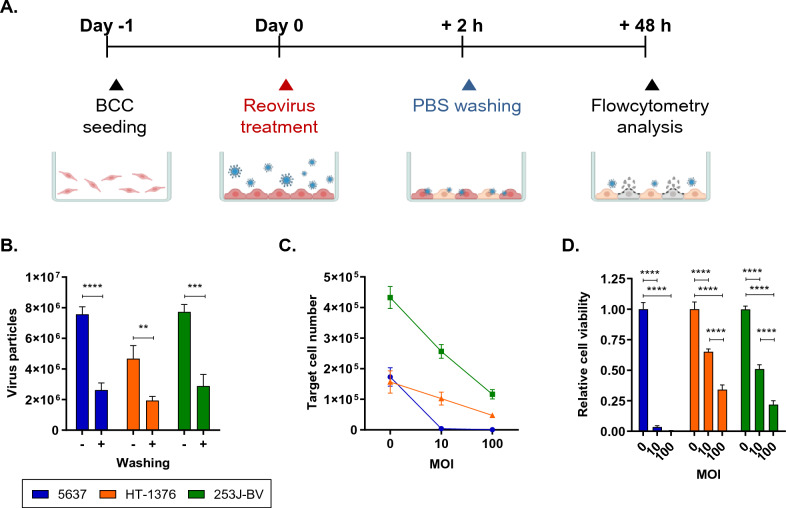
Scheme of an intravesical therapy-simulating in vitro assay and measuring the oncolytic activity of reovirus. 5637, HT-1376, and 253J-BV cell lines were treated with RP116 at an MOI of 10 or 100 MOI for 2 h, followed by a washing step with PBS. To evaluate the anti-tumor effect of RP116, the remaining live target cells were assessed after an additional 48 h of culture. (**A**) Schematic representation of the intravesical therapy-simulating in vitro assay. The supernatant was collected after an additional 2 h to measure the remaining viral particles, which were compared to those in the samples without washing. (**B**) The reduction in viral titer resulting from PBS washing was determined by quantifying the average concentration of viral particles (sized 65–75 nm). (n = 7–11). (**C**) The number of surviving target cells was observed using counting beads after RP116 treatment. (n = 6–10). (**D**) The relative viability of each cell line was analyzed. (n = 6–8) Statistical significance was calculated by Mann–Whitney test (**B**) and one-way ANOVA with Turkey’s multiple comparisons test in (**D**). **p < 0.01; ***p < 0.001; ****p < 0.0001.

In intravesical therapy, drugs injected into the bladder remain in the bladder for approximately 2 h, which restricts the duration of the direct exposure of the bladder cancer cells to the drugs. We implemented a strategy wherein bladder cancer cells were treated with reovirus, followed by washing after 2 h to control the duration of cancer cell exposure to the reovirus, mirroring the conditions of intravesical therapy. After 48 h, we assessed the number of remaining live cancer cells (Fig. 2A). The quantity of reovirus in culture media reduced after washing (Fig. 2B). However, the number of surviving cancer cells decreased in a dose-dependent manner (Fig. 2C). While a low MOI of 10 resulted in the majority of cancer cells dying in the 5637 cell line, HT-1376 and 253J-BV cell line still exhibited a substantial number of viable cancer cells, even at a high MOI of 100 (Fig. 2D). In summary, short-term reoviral treatment demonstrated cytotoxicity against all bladder cancer cell lines; however, its effectiveness varied among different cancer types.

### Evaluating cytotoxicity of eNK cells on bladder cancer cell lines

**Figure 3:**
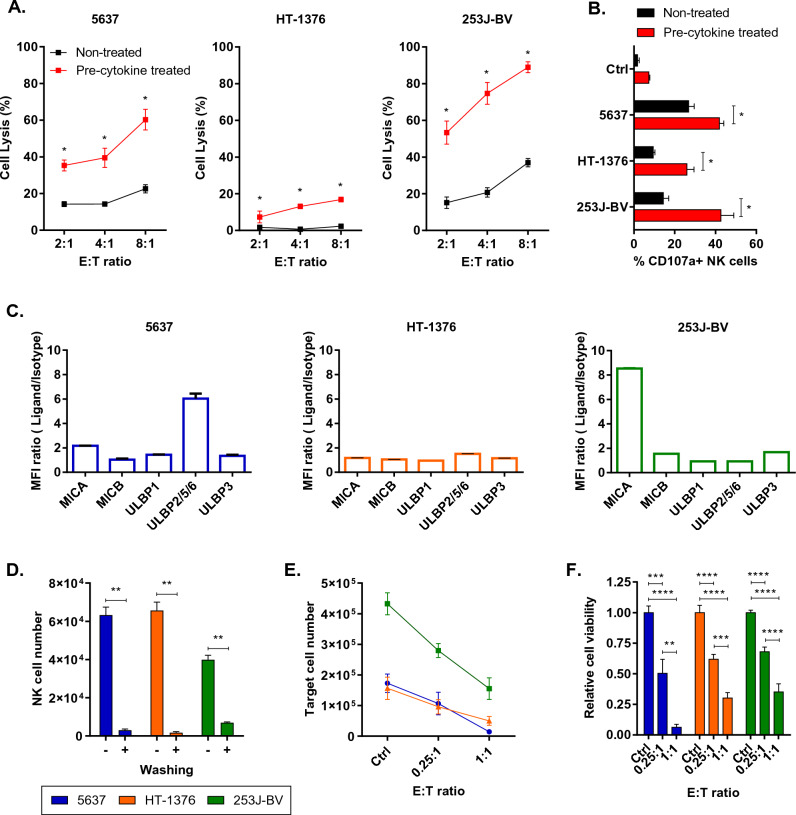
Effect of eNK cells on bladder cancer cell lines in an in-vitro assay emulating intravesical therapy. PBMC-derived NK cells were utilized in the experiments. Cytokine-pretreated eNK cells were cultured with 50 ng/mL of IL18 and 10 U/mL of IL-21 overnight before cytotoxicity assay. (**A**) eNK cell cytotoxicity against 5637, 253J-BV and HT-1376 was analyzed after 2 h after treatment with the indicated E:T ratios (n = 3) (**B**) Compiled data from three donors showing the percentage of CD107a expression (n = 3). (**C**) NK cell mediated ligands on BCC lines was detected via flow cytometry, and the results are presented as the mean fluorescence intensity (MFI) ratio based on triplicate values in a representative experiment (n = 3). (**D**) The remaining number of eNK cells was quantified after PBS washing. (n = 6) (**E**) The remaining target cell population was quantified using counting beads after eNK cell treatment (n = 6–10). (**F**) The relative viability of each cell line was analyzed (n = 6–10). Statistical significance was calculated using Mann–Whitney test (**B**–**D**) and one-way ANOVA with Turkey’s multiple comparisons test (**F**). *p < 0.1; **p < 0.01, ***p < 0.001, ****p < 0.0001.

In an ex vivo study, we explored the efficacy of eNK cells for bladder cancer, targeting three distinct bladder cancer cell lines. CFSE-labeled target cells were incubated with eNK cells, and survival was measured by PI staining. After eNK cell treatment for 2 h, 5637 and 253J-BV cells exhibited cytotoxic effect of approximately 20% and 40% (E:T ratio 8:1), respectively (Fig. 3A). However, HT-1376 cells showed cytotoxicity of only 2%. Given that NK cell killing occurs through interaction between target cells and receptors, we examined the expression of representative NK cell-mediated ligands, those recognized by the NKG2D receptor, including MICA, B and ULBP1, 2/5/6, 3, in BCC lines. We observed that 5637 and 253J-BV each exhibited high expression of ULBP2/5/6 or MICA respectively, along with other NK-mediated ligands (Fig. 3C). Thus, these findings indicate that cell lines expressing higher levels of NKG2D mediated ligands are more susceptible to the anti-tumor effects of NK cells.

Pre-treating eNK cells with IL-18 and IL-21, which are known to enhance NK cell activation, resulted in a marked enhancement of cytotoxicity when applied to BCC cell lines. Specifically, the cytotoxicity level in 5637 cells reached 60%, whereas 253J-BV cells displayed a remarkable 90%. HT-1376 cells exhibited a notable 20% cytotoxicity rate (Fig. 3A). The enhanced cytotoxicity of IL-18/-21-pretreated eNK cells was further validated by increased CD107a expression (Fig. 3B).

Finally, we followed a methodology similar to that of the reovirus experiment, which mirrored an intravesical therapy process. After exposure of BCC lines for 2 h following pretreatment of eNK cells with IL-18/-21, we performed a washing step and measured the number of viable cancer cells after 48 h. For practical application of NK cells in clinical settings, we set the maximum E:T ratio to 1:1. The results showed a significant reduction in the number of IL-18/-21-pretreated eNK cells that remained among the bladder cancer cells after washing (Fig. 3D). However, for all bladder cancer cell lines, the number of surviving cancer cells decreased based on the number of IL-18/-21-pretreated eNK cells. Approximately all 5637 cells were found dead, while more than ~ 25% of the cancer cells remained viable in the HT-1376 and 253J-BV cell lines (Fig. 3E,F). Consequently, these findings indicate that despite PBS washing, RP116 and IL-18/-21-pretreated eNK cells exhibited cytotoxicity against BCC. However, the limitations of eNK cell monotherapy became apparent, particularly in the case of HT-1376 and 253J-BV cells. To address these limitations, we devised a combination therapy involving RP116 and IL-18/-21-pretreated eNK cells.

### Examining the effect of RP116 on eNK cells

**Figure 4:**
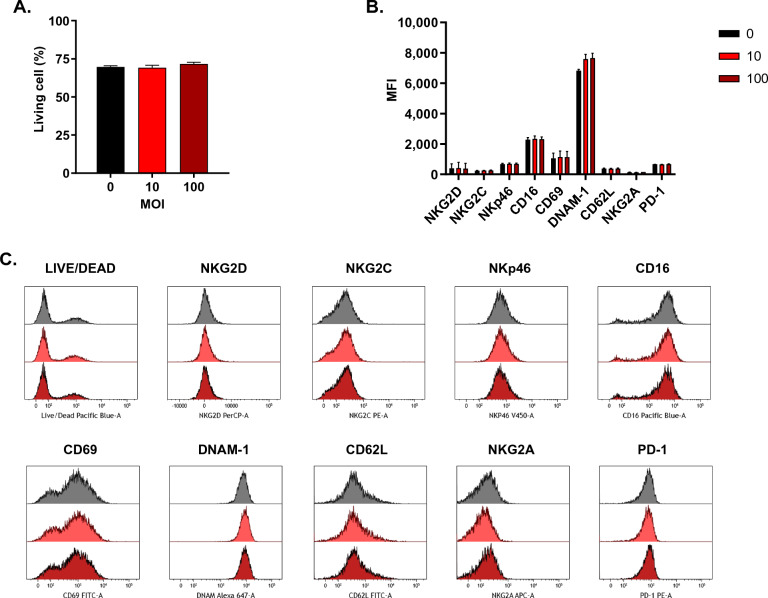
Effect of RP116 on eNK cells. The expression of NK cell receptors was analyzed by flow cytometry. IL-18/-21-pretreated eNK cells were incubated in the absence or presence of RP116 at an MOI of 10 and 100 MOI for 48 h with 10 U/mL of IL-2. (**A**) eNK cell survival was assessed using live/dead staining after RP116 treatment for 48 h (n = 3). (**B**) Compiled data from six donors showing MFI. (**C**) Representative flow cytometry plots showing the expression of eNK cell receptors. *MFI* mean fluorescence intensity.

Before administering the combination therapy of RP116 and IL-18/-21-pretreated eNK cells to BCC lines, we examined the effects of RP116 on IL-18/-21-pretreated eNK cells. After RP116 treatment, no difference in the viability of eNK cells was observed at 48 h (Fig. 4A). The expression levels of eNK receptors related to NK cell cytotoxic function (NKG2D, NKG2C, NKp46, CD16, CD69, DNAM-1, and CD62L as activating receptors and NKG2A and PD-1 as inhibitory receptors) also remained unchanged (Fig. 4B,C). These results indicate that the combination treatment with RP116 and eNK cells doesn't compromise eNK cell-mediated cytotoxicity.

### Assessing combined therapy of RP116 and IL-18/-21-pretreated eNK cells on bladder cancer cells

**Figure 5:**
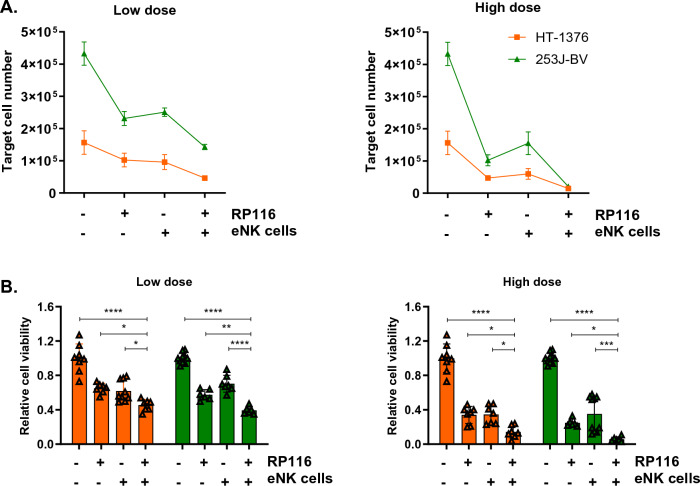
Combinational therapy of RP116 and IL-18/-21-pretreated eNK cells on bladder cancer cells. 253J-BV and HT-1376 were treated with mono-treatment of RP116 and eNK cells, as well as their combination, to evaluate the cell lysis effect. For low doses, E:T ratio of 0.25:1 and MOI 10 were utilized. For high doses, E:T ratio of 1:1 and MOI 100 were employed. (**A**) The number of living residual target cells was analyzed by counting beads after PI staining (n = 6–10). (**B**) The relative viability of each cell line was analyzed (n = 6–10). Statistical significance was calculated using one-way ANOVA with Turkey’s multiple comparisons test in (**B**). *p < 0.1; **p < 0.01, ***p < 0.001, ****p < 0.0001.

Except for the 5637 cell line, which responded well to monotherapy, we assessed the efficacy of the combination treatment on HT-1376 and 253J-BV cells, which exhibited a limited response to RP116 or IL-18/-21-pretreated eNK cell monotherapy. When treated with a relatively low dose of RP116 (MOI of 10) and IL-18/-21-pretreated eNK cells (E:T ratio of 0.25:1), the combination treatment showed the lowest number of viable cancer cells in both cell lines (Fig. 5A). However, the effect was not sufficient (< 0.5 relative viability in both cell lines) (Fig. 5B). In contrast, with a higher dose of RP116 (MOI of 100) and IL-18/-21-pretreated eNK cells (E:T ratio of 1:1), the combination treatment showed low cancer cell viability (14% in HT-1376, 6% in 253J-BV) (Fig. 5B). Taken together, the combination therapy resulted in a higher decrease in the viability of target cells compared to the monotherapy, and this effect was maximized at high doses.

## Discussion

This study demonstrates the potential of a combination therapy strategy using reovirus and NK cells for the treatment of bladder cancer. We utilized RP116, a σ1 truncated reovirus variant, and IL-18/-21-pretreated eNK cells for local high-dose injection agents in vitro assay to simulate intravesical therapy. Our findings demonstrate that the combination of RP116 reovirus and IL-18/-21-pretreated eNK cells exhibited promising additive anti-tumor effects against different grades of bladder cancer cell lines (5637, HT-1376, and 253J-BV).

Oncolytic reoviruses can be safely and repeatedly used to treat colon cancer, pancreatic cancer, and other malignancies without severe toxicities^16^. Furthermore, diverse oncolytic viruses, such as vaccinia virus, CG0070 (oncolytic adenovirus family) and T-VEC (herpes simplex virus), have been investigated in bladder cancer research (NCT04452591)^24,25^. Hence, we opted for a strategy for treating BCC cell lines with reovirus, whose clinical safety has been proven. In this study, our data demonstrated that both RC402 and RP116 exhibited anti-tumor effects against BCC lines that expressed JAM-A, and these effects increased over time, with RP116 showing particularly significant efficacy (Figs. 1 and 2). In these bladder cancer cells, it is necessary to find the difference in oncolytic activity between the two oncolytic viruses as RP116 has a truncated σ1. Given that the truncated globular head of σ1 has the function of binding to JAM-A, bladder cancer cells may exhibit a higher expression of other reovirus receptors than that of JAM-A. In fact, the high expression of sialic acids on the host cell surface can promote a high attachment to σ 1, which can lead to an increase in apoptosis of infected cancer cells^26^. Furthermore, σ 1 of reoviruses can induce cell death in infected cancer cells^10,27,28^. Hence, understanding how truncated σ1 facilitates the cell death pathway in these bladder cancer cells could offer a novel therapeutic approach for bladder cancer treatment.

In oncolytic virus therapy, several routes of administration are available, including intrathecal, intravenous, intraperitoneal, and subcutaneous^29^. Bladder cancer is commonly treated using intravesical therapy, which involves the direct application of drugs, such as BCG, into the bladder. Although this method has a brief exposure time for patients, typically around 2 h, it offers the advantage of delivering higher drug doses than systemic administration via the bloodstream. Therefore, in this study, we first established an in vitro experimental model to simulate intravesical therapy and treated the cells with a high dose of reovirus, up to an MOI of 100. However, despite the high concentration, we encountered limitations associated with reovirus monotherapy in high-grade bladder cancer cell lines, such as HT-1376 and 253J-BV.

Therefore, we explored the possibility of using a combination therapy to enhance cytotoxicity. Intravesical drug combinations involving immunotherapy and targeted therapies have emerged as novel therapeutic options for enhancing the efficacy of bladder cancer treatment and preventing recurrence^7^. In this study, we aimed to demonstrate the therapeutic effect of combination treatment with eNK cells and reovirus. eNK cells exert anticancer effects against bladder cancer^30^. In addition, genetically engineered feeder cells enable large-scale expansion of NK cells^21,31–33^. In particular, this strategy has enabled the production of off-the-shelf therapeutic agents that do not induce graft-versus-host disease (GVHD). We performed NK cell expansion using K562-mbIL-18/-21 cells, which were developed in our previous study, to assess the susceptibility of BCC to eNK cells. Furthermore, we observed an increase in the proliferation and cytotoxicity of NK cells following short-term exposure to the cytokine IL-18/-21^21^. Based on the reported findings, we used pre-treatment of eNK cells with IL-18/-21 in this study, which led to higher cytotoxicity of these eNK cells than that of the untreated eNK cells. Additionally, high E:T ratios demonstrated higher cytotoxicity against BCC cell lines than low E:T ratios. Nevertheless, we faced limitations in HT-1376 and 253J-BV cell lines in terms of monotherapy with the eNK cells or reovirus treatment. Therefore, we designed a combination therapy by integrating eNK cells, known for their safety and killing effects, with reovirus treatment.

Our data confirmed that RP116 does not influence the function of IL-18/-21-pretreated NK cells (Fig. 4). Therefore, we evaluated the efficacy of the combination of RP116- and IL-18/-21-pretreated NK cells under transient drug exposure conditions similar to intravesical therapy. To simulate intravesical therapy in our in vitro study, bladder cancer cell (BCC) lines were treated with RP116, IL-18/-21-pretreated expanded NK (eNK) cells, or a combination of both for 2 h, followed by washing with PBS.

The findings demonstrate a substantial decrease in the population of IL-18/-21-pretreated eNK cells, with fewer than 1 × 10^4^ cells persisting in association with bladder cancer cells after the washing procedure (Fig. 3E). However, virus particles of RP116 were approximately one-third of that in the control group (Fig. 2B). These findings suggest that during the additional 2 h of incubation, newly produced RP116 might have originated from target cells that underwent damage or that RP116 bound to reovirus receptors present on the target cells could have been evaluated (Fig. 2B). Finally, we observed dose-dependent anti-tumor effects of RP116 and eNK cells on the three types of BCC lines, with 5637 cells exhibiting the highest sensitivity to both mono-treatments (Figs. 2C,D, 3E,F). Furthermore, we confirmed an additive effect when RP116 and eNK cells were used in conjunction, as they exhibited increased cytotoxicity compared to mono-treatments, without adversely affecting each other's function. Based on these results, a combination therapy with reovirus and eNK cells could be considered as an adjuvant treatment option for bladder cancer after resection, providing the potential to impede disease progression even if complete eradication is not achieved.

In this study, we designed an in vitro experiment to simulate the intravesical therapy used in clinical settings for treating bladder cancer, however, there are limitations to our research. Firstly, we were unable to validate our findings through animal studies, which could provide further insights into the therapy's efficacy and safety profile. In clinical research on intravesical therapy, the drug effect is evaluated by assessing the absorption rate within the bladder tissue and the residual drug concentration in the urine after excretion^34,35^. Post-PBS washing, we quantified eNK cells by identifying CFSE-negative cells using flow cytometry, and RP116 levels were measured with Nanosight, a device capable of quantifying virus concentrations^36,37^. The residual RP116 and IL-18/-21-pretreated eNK cells showed anti-tumor effects on BCC (Figs. 2C,D, 3E,F). However, it is difficult to verify the additional effects from reovirus or eNK cells that attached to cancer cell lines, as we only measured single-cell NK states or intact-sized RP116 (70–100 nm). Therefore, an in vivo experiment is necessary to confirm the potential effects and safety of the residual RP116 and eNK cells, and it would further solidify the suitability of our in vitro assay.

Secondly, the actual clinical environment encompasses a variety of immune cells, whereas our study primarily utilized eNK cells, thereby limiting our ability to explore interactions with other immune cells. This discrepancy could lead to differences between our in vitro results and clinical outcomes. Reoviruses induce antitumor activity by activating immune cells, such as dendritic cells (DC) and T cells^38–40^. Nonetheless, our research revealed that the reovirus did not affect the expression of NK cell receptors (Fig. 4B,C) or had any significant effect on the expression of NKG2D-related ligands (MIC and ULBP families) in the BCC lines (data not shown). Furthermore, we tested the sequential administration of RP116 and eNK cells; however, no significant difference in their efficacy was observed when NK cells were added after RP116 treatment, or vice versa. This phenomenon may be attributed to the absence of peripheral immune cells, such as DCs and antigen-presenting cells, which can serve as signaling intermediaries. Taken together, studying the interaction between infused reoviruses and NK cells in the bladder and other immune cells in patients could lead to an improved therapeutic approach with the potential to effectively treat bladder cancer.

## Materials and methods

### Ex vivo expansion of NK cells

NK cells were expanded from peripheral blood mononuclear cells (PBMCs) via co-culture with 100 Gy gamma-irradiated K562‑mbIL‑18/-21 cells, as described previously with minor modifications^31,41,42^. Briefly, healthy donor-derived human PBMCs were isolated by density-gradient centrifugation using Ficoll-Hypaque (d = 1.077, LymphoprepTM; Axis-Shield, Oslo, Norway). PBMCs were co-cultured with irradiated (100 Gy) K562-mb-IL-18/21 cells with RPMI 1640 medium supplemented with 10% FBS, 100 U/mL penicillin, 100 µg/mL streptomycin, and 4 mmol/L l-glutamine. The culture medium contained 10 U/mL recombinant human IL-2 (PeproTech, Rocky Hill, NJ, USA). After day seven, the concentration of IL-2 was increased from 10 to 100 U/mL, and 5 ng/mL of soluble IL-15 (PeproTech) was added to the medium. The medium was refreshed every two–three days. To minimize phenotypic changes, NK cells were used after day 14 and within one month. This study was approved by the Institutional Review Board of the Samsung Medical Center, Seoul, Korea (IRB No. SMC 2021-07-155-002). Pre-cytokine-treated NK cells were stimulated with 50 ng/mL of IL-18 (MBL International, Woburn, MA, USA) and 10 U/mL of IL-21 (PeproTech) one day prior to the experiment.

### Reovirus, cell lines, and culture conditions

The bladder cancer cell lines 5637 and HT-1376 were purchased from the American Type Culture Collection (ATCC). 253J-BV was purchased from the Korean Cell Line Bank. The cell line 5637 was cultured in RPMI-1640 (Hyclone Laboratories, Logan, UT, USA) supplemented with 10% fetal bovine serum (FBS, Gibco) and 1% (v/v) penicillin/streptomycin (Hyclone). HT-1376 and 253J-BV cells were cultured in DMEM supplemented with 10% fetal bovine serum (FBS, Gibco). All cells were maintained at 37 °C in a humidified atmosphere containing 5% CO_2_. Wild-type Reovirus T3D (RC402) and a variant of RC402 (RP116) were obtained from ViroCure, Inc. The RC402 cells were purchased from the Korea Bank for Pathogenic Viruses (KBPV). RP116 is an attenuated reovirus that is naturally generated by infecting HT1080 human fibrosarcoma cells with wild-type reovirus and cultivating them for an extended period^10^. Reoviruses were stored at a temperature of – 80 °C until further use.

### Antibodies and flow cytometry

The following fluorophore-conjugated antibodies were used for BCC lines and NK cell staining: anti-hCD321 (JAM-A; clone OV-5B8) were derived from BioLegend (San Diego, California, USA). anti-hMICA (clone 159227), anti-hMICB (clone 236511), anti-ULBP1 (clone 170818), anti-ULBP2/5/6 (clone 165903), anti-ULBP3 (clone 166510), anti-CD3 (clone SK7), anti-CD56 (clone CMSSB), anti-CD16 (clone CB16), anti-CD69 (clone FN50), anti-NKG2D (clone 1D11), anti-NKp46 (clone 9E2), anti-PD-1 (clone MIH4), and anti-CD62L (clone DREG56) were derived from Invitrogen (Waltham, Massachusetts, USA); anti-NKG2A (clone 131411) and anti-NKG2C (clone 134591) were derived from R&D systems (Minneapolis, Minnesota, USA); anti-DNAM-1 (DX11), and anti-CD107a (clone H4A3) were derived from BD bioscience (San Jose, CA, USA). The Live/Dead Fixable violet dead cell stain kit (Invitrogen) was used to determine the NK cell viability. The cells were then stained with antibodies for 30 min on ice. Flow cytometry was performed using a FACSVerse or FACSLyric (BD Biosciences). Flow cytometry data were analyzed using the Kaluza software (Beckman Coulter Inc., Brea, California, USA).

### Cell viability assay

A total of 5 × 10^3^ 5637, HT-1376, and 253J-BV cells were seeded in a 96-well plate in triplicate. The cells were exposed to reoviruses at a multiplicity of infection (MOI) of 0–100. After 24, 48 and 72 h, the viability of cells was determined using CellTiter 96® AQueous One Solution Cell Proliferation Assay (Promega, Madison, Wisconsin) according to manufacturer’s instruction.

### Intravesical therapy condition-simulating in vitro assay

Cytotoxicity of eNK cells against BCC lines (5637, HT-1376, 253J-BV) was measured by carboxyfluorescein diacetate succinimidyl ester (CFSE; Life Technologies)-based assay for 4 h. Target cells were stained with 0.5 μM CFSE in FACS buffer for 10 min at 37 °C and washed twice with complete media. The target cells were then placed in 24-well treatment plate at a density of 5 × 10^4^ one day before treatment. The target cells were treated with mono- or combination treatment with RP116 and eNK cells at indicated MOI and E:T ratios. The cells were incubated at 37 °C in an incubator containing 5% CO_2_ for 2 h. The wells were then washed with PBS and treated with new completed media. After 48 h, the absolute number of living target cells was determined using CountBright absolute counting beads (Invitrogen) stained with propidium iodide (PI; Sigma-Aldrich, St. Louis, MO, USA).

### CD107a degranulation

The eNK cells (5 × 10^3^) were incubated with or without BCC lines (5637, HT-1376, 253J-BV) at a density of 5 × 10^3^ in a 96-well treatment plate in the presence of anti-CD107a (5 μL). Following 1 h of incubation, monensin and brefeldin A were introduced, and the plate was further incubated for 4 h. NK cells were stained with anti-CD3 and CD56 mAbs before acquisition.

### Quantification of residual virus particles and NK cell numbers

BCC cell lines (5637, HT-1376, and 253J-BV) plated in 24-well plates were treated with RP116 (MOI 100) or eNK cells (1:1 E:T ratio) for 2 h. For mock control, the wells were incubated without further manipulation. The wells in the mono-treatment were replaced with new completed media after washing with PBS. Following additional 2 h incubation, supernatants were collected, while the cells adherent to the well were detached using Trypsin–EDTA (Gibco). The average concentration of viral particles with sizes ranging from 65 to 75 nm in the supernatant was estimated using a NanoSight NS300 (Malvern Panalytical).

### Statistical analysis

Statistical analyses were performed using the GraphPad Prism 6 software (GraphPad Software, San Diego, CA, USA). For normally distributed variables, comparisons between two groups were performed using the Mann–Mann–Whitney test, and multiple group comparisons were determined using one-way ANOVA. Significant differences were defined as *p < 0.05, **p < 0.01, and ***p < 0.001.

## Data Availability

The data that support the findings of this study are available from the corresponding author upon reasonable request.
